# Presence of Histatin-1 in Human Tears and Association with Aqueous Deficient Dry Eye Diagnosis: A Preliminary Study

**DOI:** 10.1038/s41598-019-46623-9

**Published:** 2019-07-16

**Authors:** Sushma Kalmodia, Kyung-No Son, Dingcai Cao, Bao-Shiang Lee, Bayasgalan Surenkhuu, Dhara Shah, Marwan Ali, Arun Balasubramaniam, Sandeep Jain, Vinay Kumar Aakalu

**Affiliations:** 1 Department of Ophthalmology and Visual Sciences, University of Illinois at Chicago, Chicago, USA; 2 Research Resources Center, University of Illinois at Chicago, Chicago, USA

**Keywords:** Diagnostic markers, Translational research

## Abstract

The aims of this study were to determine if histatin-1 (H1) is present in normal human tears and whether tear levels of H1 varied between normal patients and those with aqueous deficient dry eye disease (ADDE). Patient samples were obtained from 11 normal patients and 11 severe ADDE patients. Relevant patient characteristics, including age, sex, and dry eye disease (DED) diagnostic parameters were collected. Multiple qualitative and quantitative methods were used to compare the concentration of H1 between patient groups. Mixed linear modeling was used to compare H1 levels between groups, and diagnostic performance was assessed using the receiver-operator-characteristic (ROC). ADDE patients had significantly lower H1 concentrations (85.9 ± 63.7 ng/ml) than the normal group (891.6 ± 196.5 ng/ml) (p < 0.001), while controlling for age and sex. ROC analysis indicated that H1 concentration is potentially a biomarker for ADDE (area under curve = 0.96). Reclassification of patients by DED parameters including, Ocular Surface Disease Index (OSDI) (≤13, >13) and Schirmer I (without anesthesia) (<10 mm, ≥10 mm) showed significant differences in H1 level (OSDI, p = 0.004) and Schirmer I ((p = 0.010). In conclusion, this is the first preliminary report of the presence of H1 in human tears. H1 concentrations are lower in ADDE patients and H1 may have diagnostic potential in evaluation ADDE patients.

## Introduction

Dry eye disease (DED) is a common disorder that affects millions of patients^1^. Aqueous deficient DED (ADDE) is associated with lacrimal gland dysfunction and loss of the aqueous layer of tears^2^. Several causes for ADDE include, but are not limited to Sjögren’s syndrome (SS) and ocular Graft-versus-Host Disease (oGVHD). Loss of the aqueous layer of the tear film can be associated with ocular surface inflammation, tear hyperosmolarity and a reduction of a number of intrinsic tear components, like lacritin^2,3^. Characterizing which components of tears are lost in ADDE can inform our understanding of the pathophysiology of disease, advance development of diagnostic measures and potentially support rational therapeutic replacement of diminished components of the tear film.

Histatins are an important class of endogenous anti-microbial peptides (AMP)^4^. Other exemplary AMPs include LL-37 and β-defensins^5^. Histatin peptides are a histidine rich family of 12 peptides (histatin 1–12), arising from two genes HTN1 and HTN3^6^. Histatins were first described as anti-fungal agents in the saliva but have since been found to have anti-viral, anti-bacterial, wound healing and even anti-inflammatory activities^6,7^. The most common variants of histatin peptides in saliva are H1, H3 and H5 and typically represent 20–30% of the total salivary histatin pool^6,8^. In whole saliva, the concentration of H1 without stimulation was 12.9 ± 3.7 µg/ml^9^. In saliva, H2, H4, H6–H12 are formed as proteolytic fragments of H1, H3 and H5^10^. Saliva of patients with rheumatoid arthritis with oral sicca symptoms has been noted to have decreased levels of histatins, and there has been interest in testing the use of histatins as markers of disease^11–13^.

Recently, there has been some interest in the potential role of histatin peptides in the ocular surface and the lacrimal functional unit. Some authors have found presence of expression of histatins in components of the ocular surface and on Schirmer tear strip samples from patients^14–16^. It was also demonstrated that H1 is present in epithelia of accessory lacrimal glands of humans, corneal and conjunctival epithelia, and that H1 can promote migration of human corneal epithelia^17,18^.

H1 is thought to be able to promote epithelial migration, adhesion, barrier integrity and reduces the effects of epithelial-mesenchymal transition promoting agents^19^. Such characteristics, if ascribed to an intrinsic component of the tear film would be important to a healthy ocular surface.

Given the increasing evidence that histatin peptides are present in the ocular surface and tear film unit and the evidence that H1 can promote epithelial wound healing^17^. We sought to test two questions:

- Do the tears of normal patients contain H1?
- Do the tears of patients with ADDE have diminished levels of histatins?.

In this study, patients were considered to have ADDE if their Schirmer I at 5 minutes was less than 10 mm of wetting, vital dye staining of the cornea ≥1 on the National Eye Institute-Corneal Staining Score (NEI-CSS), and an OSDI score greater than 13^20,21^. We focused primarily on H1 as this is one of the most studied in saliva, has been demonstrated in the lacrimal functional unit, and could be an interesting tear component to replace due to its epithelial trophic functions^17,22^.

## Results

### Patient demographics and phenotypic characteristics

Patient demographic, clinical data and statistical comparisons are shown in Tables 1 and 2. Compared to the normal group, the ADDE group had a higher age (53.5 yrs. ± 2.9 yrs. vs. 44.6 yrs. ± 2.6 yrs., p = 0.032 from t testing) and a comparable percentage of males (54.6% vs. 63.6%, p = 0.665 from Chi-square testing). Consistent with our clinical definition of ADDE, we found that the ADDE group had a significantly lower Schirmer I measurements [1.9 ± 0.8 mm vs. 18.3 ± 2.5 mm, p < 0.001 from t-testing] and higher OSDI score (33.8 ± 6.6 vs. 1.9 ± 1.0, p < 0.001 from t-testing) than the normal group (Table 1). With an average Schirmer I of 1.9 mm, our ADDE patient population consisted of severe disease phenotypes. Additional phenotypic data including: non-invasive tear break up time (NITBUT), NEI-CSS and Meiboscale scores^23,24^ are included in the Supplemental data section (Supplementary Table 1). Notably, NITBUT [(secs), p = 0.392] and NEI-CSS (0–15), p = 0.3045) were not significantly different between ADDE subgroups (SS and oGVHD) whereas, Meiboscale score (0–4) means were significantly different between SS and ADDE subgroups (SS, 0.4 ± 0.2 and oGVHD 1.1 ± 0.1, p = 0.0202) (Supplementary Table 1).

**Table 1:** Patient Characteristics and Histatin-1 Concentrations.

Risk Factors	Normal (N = 11) Mean(se) or %	ADDE (N = 11) Mean(se) or %	p-value
Age (yrs)	44.6 ± 2.6	53.5 ± 2.9	0.032
Male Sex	7(63.6%)	6(54.6%)	0.665
Schirmer I (mm)	18.3 ± 2.5	1.9 ± 0.8	<0.001
OSDI	1.9 ± 1.0	33.8 ± 6.6	<0.001
HI (ng/ml)	891.6 ± 196.5	85.9 ± 63.7	<0.001

Schirmer I = Testing, in 5 minutes.

ADDE = aqueous-deficient dry eye.

OSDI = Ocular Surface Disease Index.

H1 = Histatin-1.

se = standard error of the mean.

% = Percentage.

**Table 2:** Risk Factors for ADDE^†^.

Risk Factor	Odds Ratio	Standard Error	p-value
H1 (100 ng/ml increase)^*^	0.5	0.1	0.016
Age (1 year increase)	1.1	0.1	0.055
Male sex	0.7	0.6	0.665

^*^H1 = Histatin-1, the mean value between two eyes for each subject was used for odds ratio calculation.

^†^ADDE** = **Aqueous-deficient dry eye, OSDI and Schirmer I testing were not included in this analysis as they were used for disease status classification. The dependent variable is disease status (ADDE vs normal) and the independent variable is H1 with age as the control variable.

### Histatin-1 is present in normal human ocular surface washings

**Figure 1:**
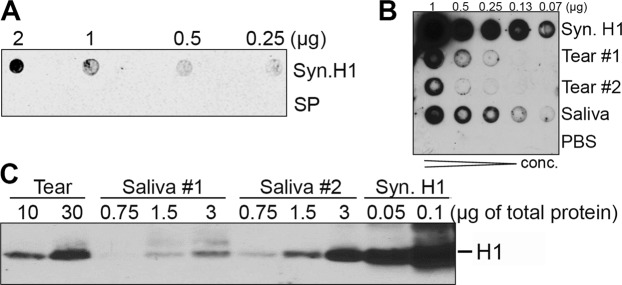
Detection of H1 in Human Tears. (**A**) Testing of mouse anti-human H1 antibody using Immune Dot Blot (IDB) of serial dilutions of synthetic H1 (Syn. H1) and scrambled peptide (SP). Notable is the lack of signal in SP and positive signal in Syn. H1, consistent with good, specific detection of Syn. H1. (**B**) Detection of H1 in saliva and tears. IDB was used to test serial dilutions of positive control (Syn. H1), biological positive control (saliva), negative control (phosphate buffered saline (PBS)) and tears of two normal controls and the experimental tear samples using rabbit anti-human H1. (**C**) Confirmation of H1 in saliva and tears using Western Blot (WB). Results demonstrate the presence of a band at the appropriate size for H1 detected by anti-H1antibody in tears of a normal patient, saliva of two normal patients and Syn. H1 using rabbit anti-human H1.

Antibody specificity was confirmed using Immune Dot Blot (IDB) over a series of concentrations of synthetic H1 (Fig. 1). Notably synthetic H1 peptide was detected with greater dot density with increasing concentrations of peptide in solution. Scrambled peptide (SP) negative control was found to be undetectable (Fig. 1A). The presence of H1 in normal human tears was then assessed using IDB and Western Blot (WB) and found that H1 peptide is present in ocular surface washings (OSW). Positive control (Synthetic H1(Syn.H1)) and biological positive control (Saliva) showed similar results (Fig. 1B,C). A full-length WB image is shown in Supplementary Fig. S1. It should be noted that the IDB in Fig. 1A,B were performed at separate times and as such comparison of signal intensity between these experiments is not possible.

**Figure 2:**
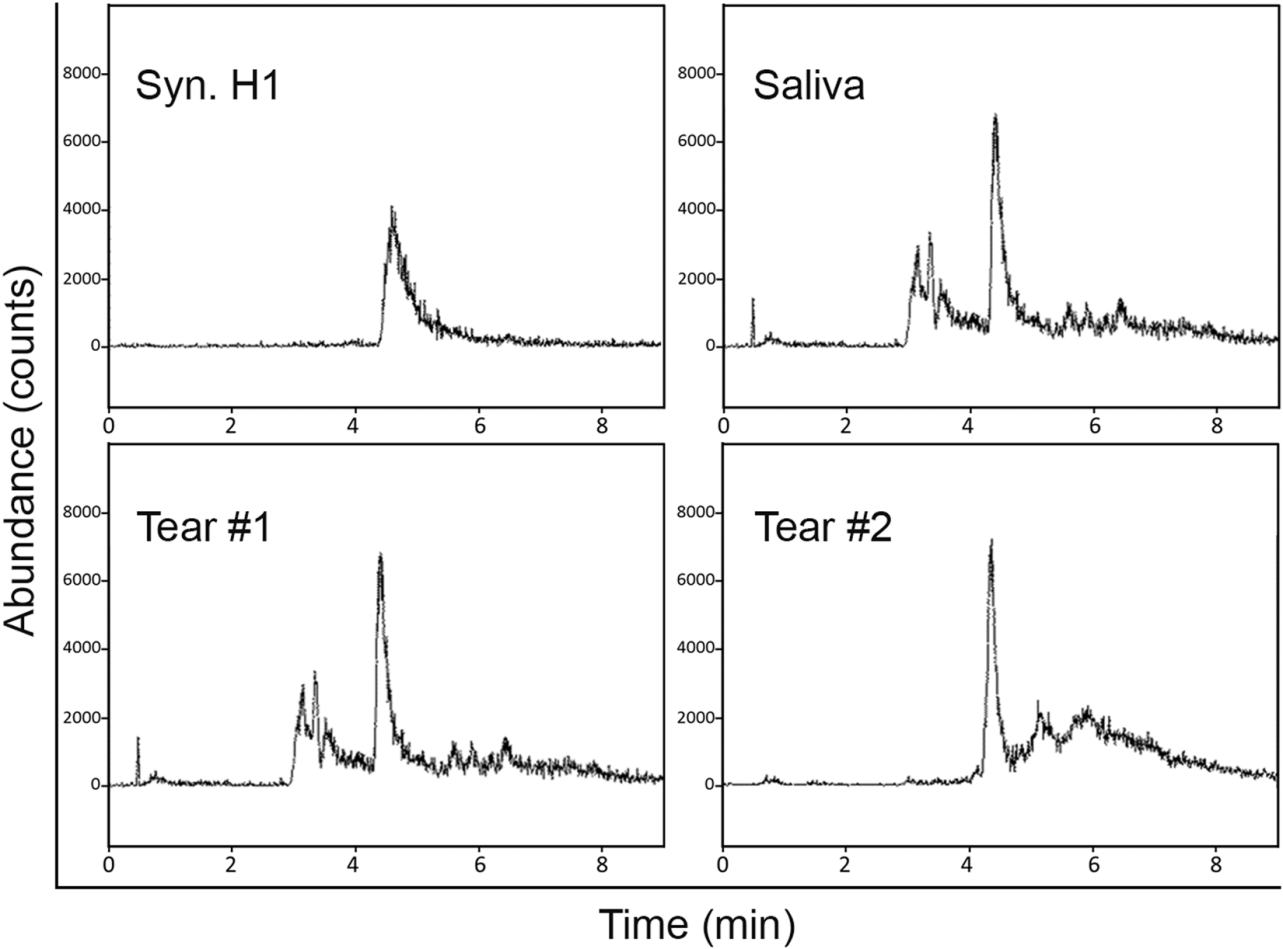
Confirmation of H1 in Human Tears using    Multiple reaction monitoring (MRM). The MRM was operated in the positive ion mode monitoring the 704.9–931.0 m/z transitions for H1 to identify the presence of H1 in saliva, tears and a positive control solution containing synthetic H1. Shown are representative results of MRM testing of tears of two normal (non DED) patients. Similar results were found with MRM testing in three separate experiments.

Multiple reaction monitoring (MRM) was then used to identify the presence or absence of H1 in OSW and to confirm or deny the WB and IDB results showing the presence of H1 in OSW, as this technique is not dependent upon antibody specificity. Targeted MRM detected the presence of H1 in solubilized Syn.H1, normal human  tear/OSW and saliva (Fig. 2).

### Histatin-1 levels are lower in ADDE patients than normal controls

**Figure 3:**
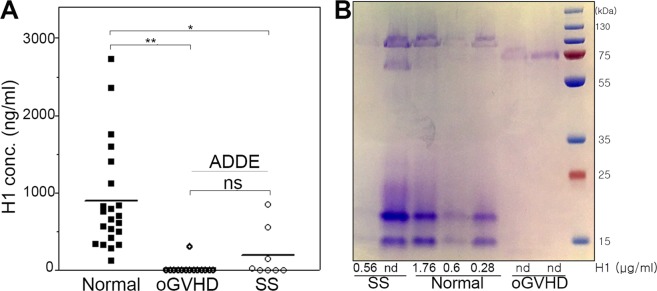
ELISA and Protein Level Comparison of Human Tear Samples for H1 Concentrations. (**A**) ELISA analysis of human tear samples/OSW of normal, SS and oGVHD patients using a commercially available H1 ELISA kit (MBS2022124 H1, Mybiosource). H1 concentration is represented in ng/ml and each measurement (each eye) is represented by a square (normal), diamond (oGVHD) or circle (SS). The mean concentration is noted by the black bar. The mean ELISA H1 for normal, all ADDE (oGVHD + SS), oGVHD and SS were 891.6 ± 196.5 ng/ml, 85.9 ± 63.7 ng/ml, 22.0 ± 22.0 ng/ml and 197.6 ± 170.2 ng/ml respectively. Statistical comparisons are noted and demonstrated statistically significant p < 0.05differences between normal and all ADDE patients, but no significant (ns) difference between oGVHD and SS. (**B**) Association of total tear protein with H1 concentration using Coomassie staining of tear proteins separated by gel electrophoresis. H1 concentrations for each patient, representing samples in each lane, were quantified by ELISA and concentrations from ELISA are noted below the lane for each patient in µg/ml (nd = not detected). Density of the staining represents the relative amount of total proteins present in the tears of each sample. Notably, there did not seem to be a correlation between total protein levels and H1 concentrations within each group and across all groups, indicating these may vary independently.

OSW were tested using enzyme-linked immunosorbent assay (ELISA) from ADDE patients and normal controls (Fig. 3A). Random intercept mixed linear modeling with eyes nested in patients indicated that the diseased group had lower H1 concentrations than the normal group [85.9 ± 63.7 vs. 891.6 ± 196.5, β(se) = −805.7(206.5), p < 0.001]. Histatin level was significantly associated with age [β(se) = −36.1(11.5), p = 0.002], OSDI score [β(se) = −13.4(5.5), p = 0.016], Schirmer [β(se) = 20.9(10.00), p = 0.036]. H1 level did not depend on sex [β(se) = −255.2(272.8), p = 0.349]. To address the association with age, mixed linear modeling was performed to control for age and diagnosis of ADDE and found that H1 association with diagnosis was still significant [β(se) = −612.0(217.4), p = 0.005].These results indicate that the difference in H1 concentrations between the diseased and normal subjects could not be accounted for by the differences in age.

In order to understand if estimates of total protein levels varied with or independently of H1 levels, (Fig. 3B) outliers (high and low H1 concentration of samples with available residual tears for testing in each group- normal, oGVHD, SS) were selected and evaluated by performing gel electrophoresis and Coomassie staining. The results of these tests showed, in general, that there was no strong relationship between total protein levels and H1 concentration, indicating they may vary independently of one another.

**Figure 4:**
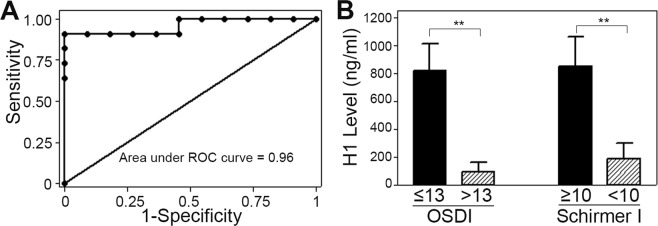
Statistical Analysis of H1 Concentration and Association with OSDI and Schirmer I. (**A**) ROC analysis of the sensitivity and specificity of H1 concentrations for detecting a diagnosis of ADDE in the tested population. Area under the ROC curve was 0.96 (95% CI = 0.87–1.00), consistent with the excellent ability of H1 concentration to be used to detect a diagnosis of ADDE in the studied population. (**B**) Testing the Ability of H1 Concentration to Detect Patients with High OSDI or Low Schirmer I values. Patients were reclassified to groups based on their OSDI scores and Schirmer I measurements. Based on OSDI classification, the H1 level was significantly lower in the high OSDI (“diseased”) than the normal group. Based on Schirmer I classification, H1 level was significantly lower in the low Schirmer I (“diseased”) than the normal group.

Receiver-operator-characteristic (ROC) analysis was then performed, which indicated that using ELISA H1 to classify ADDE status yielded an area under of curve of 0.96 (95% CI = 0.87–1.00) (Fig. 4). By using a cutoff with ELISA H1 ≤378 ng/ml as a criterion for ADDE, we obtained a sensitivity of 90.9% and specificity of 90.9% for ADDE diagnosis. These results indicate that H1 levels have potential diagnostic use for ADDE. Given our findings that ADDE diagnosis, as defined by clinical criteria noted in the methods strongly associated with H1 levels, we sought to evaluate whether H1 levels were associated with specific diagnostic elements. The association between H1concentration and disease was then assessed by reclassification of subjects into DED defining criteria: OSDI and Schirmer I. Based on OSDI classification, the H1 level was significantly lower in the diseased group than the normal OSDI group (ADDE: 94.5 ± 69.8 ng/ml vs. normal: 817.3 ± 194.1 ng/ml, t(20) = 3.2, p = 0.004) (Fig. 4). Similarly, based on Schirmer I classification, H1 level was higher in the normal group than the normal Schirmer I group (ADDE: 187.1 ± 116.7 ng/ml vs. normal: 850.7 ± 212.5ng/ml, t(20) = 2.9, p = 0.010).

## Discussion

The main findings of this study are that H1 is present in human tears and levels of H1 are significantly reduced in patients with ADDE. This study represents the first robust demonstration of H1 in human tears, building upon initial findings amongst multiple groups^14,15,17,22^.

Multiple qualitative and quantitative methods were used to confirm the presence of H1 in OSW. H1 was demonstrably present in human OSW using Immune Dot Blot (IDB), western immunoblotting, ELISA and MRM. Moreover, H1 expression level was found to vary significantly between normal and ADDE patients. This demonstration of the presence of histatin in tears, and deficiency of H1 in ADDE, suggests that evaluation of H1 in the pathogenesis of disease and utility in diagnosis and therapy of ADDE merit further investigation. The potential importance of histatins in ocular surface biology is highlighted by their demonstrated importance as contributors to oral epithelial and salivary homeostasis. Additionally, the importance of histatins levels as potential diagnostics is shown by findings lower than normal salivary histatin levels in rheumatoid arthritis patients with oral sicca symptoms^11,13^.

Interestingly, previous non-targeted proteomics studies using traditional LC-MS methods have not demonstrated the presence of H1 in human tears^25^. It is possible H1 was not previously detected by LC-MS because of the limitations of traditionally used methodology and the superiority of MRM in directed testing for specific proteins in human bodily fluids^26,27^. Clinical phenotype of DED also seems to have a considerable effect on tear protein composition, with aqueous deficiency associated with greater alterations of protein expression patterns. Additionally, it should be noted that not many proteins appear to be selectively downregulated in dry eye disease, highlighting the importance of these altered proteins as potential markers or treatments of disease^28^. Moreover, variations in total protein level in tears did not correlate with levels of H1, suggesting that H1 concentrations vary independently of protein concentration in tears in general.

We found that H1 levels are lower in ADDE patients than normal patients. We also found that there was a negative association with age and H1 levels. After controlling for age, using statistical modeling, the association between H1 and ADDE diagnosis was still present, suggesting that age differences do not account for the association of low H1 levels and ADDE diagnosis. There was also a strong correlation of H1 concentration with gold standard diagnostic measures of DED, including OSDI and Schirmer I values. Post-hoc pairwise comparisons between subgroups demonstrated significantly different H1 concentrations between normal and oGVHD (p = 0.002), significant differences between normal and SS (p = 0.026) and no significant difference between SS and oGVHD (p = 0.576). It should be noted that this study was not designed to compare these subgroups and future, larger, studies will be needed for comparison of H1 levels in different subtypes of ADDE.

In the studied patient population, it was noted that H1 as a risk factor for having a diagnosis of ADDE, with a higher odds-ratio (OR) was stronger (OR = 0.5) than age (OR = 1.1) or sex (OR = 0.7) (Table 2). Specifically, it was noted that, for every increase in 100 ng/ml of H1, there was a 0.5 reduction in risk of ADDE. Also, of note is the strong association between H1 level and OSDI (Fig. 4B), which can sometimes vary independently of objective measures of tear deficiency^29^. Future studies to compare H1 levels of patients that have symptomatology of DED, without other objective measures, the so-called “pain without stain” patients would be of interest.

The presence of H1 in human tears and the decrease of H1 in patients with ADDE open several potential areas of inquiry. One, what is the physiologic importance of H1 in human tears and the tear functional unit as a whole? It has been reported that H1 is present in lacrimal epithelia, and that histatin peptides can enhance corneal epithelial migration^17,30^. Two, can H1 serve as a biomarker for ADDE? This would have to be investigated in future studies looking at different subtypes of ADDE with larger variation in severity. Additionally, changes in H1 levels in response to treatment of DED using anti-inflammatory or other treatment options would be of interest to investigate the use of H1 as a biomarker. Three, given the presence of H1 in tears and the reduction of these levels in ADDE, is there value to replacement of H1 in patients with ADDE? As noted prior, histatin peptides are critical components of the oral mucosal environment, playing important roles in wound healing, inflammation abrogation, epithelial trophism, and antibiosis^31^. Similarly, H1 can affect potential derangements seen in DED in corneal epithelia by enhancing migration^30^. Other authors have recently reported on the utility of histatin peptides in treating corneal epithelial wounds in rabbits^32^. Future studies assessing the effects of H1 replacement in models of ADDE or other relevant ocular surface diseases would be of considerable interest. Moreover, establishment of the mechanisms of action of histatin peptides on corneal epithelia and other ocular surface tissues should be investigated.

This study should be considered in the context of increasing evidence that endogenous anti-microbial and other endogenous proteins contribute significantly to the immune and environmental responses and homeostasis of the ocular surface. There are multiple examples of important endogenous peptides, that change in disease states. For instance. human beta-defensin2 is expressed in conjunctival epithelial cells and increases in DED patients. Another example is the observed reduction of lipocalin and lacritin expression in DED patients^33–36^. These types of associations have pathophysiologic importance, as is seen in the changes in surface tension and viscosity of the tear film with alterations in lipoaclin expression. Lacritin, like histatin, is also noted to have numerous salutary effects on ocular epithelia^17,35^. Given the importance of these peptides and their derangement in DED, it is possible that divergence from normal physiologic peptide levels could affect ocular surface epithelial function. Future studies demonstrating dysfunction in ocular surface epithelia associated with abnormal peptide levels would also support the utility of restoring normal peptide levels, as a potential therapeutic approach to disease^33–37^.

There are several limitations to consider in evaluating this preliminary study, including limited sample size, inclusion of primarily severe ADDE patients and minor technical limitations. Although, this study had a relatively small sample size (n = 22) and imperfect age matching, it is closely comparable to several recent studies examining various protein levels in tears of patients (which ranged from 3 patients per group to 25 patients per group)^12,38–43^. To address lack of age matching statistical methods were used to control for this issue, but cannot completely substitute for a perfectly matched study. Ideally, normative levels in a large group of patients would be collected prospectively in an independent confirmatory study. Additionally, the bulk of our tested ADDE patients had severe disease. In order to better evaluate the relevance of H1 levels in DED as a whole, multiple severities and types of DED should be tested. In order to evaluate the utility of H1 levels as biomarkers of disease, it will also be necessary to test concentrations in response to treatment. Additionally, testing of the association other potential biomarkers including Matrix metallopeptidase 9 (MMP-9) and hyperosmolarity with histatin levels would be of interest to determine the value of H1 levels as markers of ADDE or DED in general. All of these issues require a larger, prospectively designed and powered study with better age matching of groups. Finally, testing of other types of histatin peptides in tears will be of interest to see if different histatins vary with disease status or other patient characteristics^12^. Technical limitations of this study include qualitative measurements of total protein in tears using Coommassie blue staining, which is inferior to other methods such as the REVERT™ Total Protein Stain (LI-COR Biosciences, Lincoln, NE) stain. Moreover use of Coomassie staining to give relative amounts of protein in tear samples has been used in other publications and indicated that the staining of gels for this purpose gave a mid-range discriminative power^44^. Given the small amount of sample available to us and the use of OSW as a source, we are unable to give quantitative data for total protein levels. Nevertheless, H1 was found to be mostly low in all ADDE patients whereas total protein was varied.

In conclusion, this study demonstrates that H1 is present in normal human tears and vary differentially between normal patients and those with ADDE. The results of this study also showed that H1 is a potentially useful diagnostic test, as H1 levels correlated well with gold standard diagnostic measures including OSDI and Schirmer I. Given the numerous biological functions of histatin peptides, including epithelial trophic, wound healing, anti-microbial and immunomodulatory actions, which are targets of ideal ocular surface disease treatments, and their reduction in ADDE, it would be reasonable to hypothesize that histatins may play a role in ADDE pathophysiology. Future studies focused on this hypothesis are planned.

## Methods

### Subject participants and classification

This research study was conducted under a University of Illinois at Chicago Institutional Review Board approved protocol, adhering to the tenets of the Declaration of Helsinki and the Health Insurance and Portability and Accountability Act. Symptomatic patients with DED and asymptomatic healthy control subjects were enrolled, and informed consent was obtained from all participants after the nature and possible consequences of the research were explained.

Participants were divided into the following two groups: (1) normal controls (n = 11, 22 eyes) (2) ADDE (n = 11, 22 eyes), with two subgroups: (a) SS group (n = 4, 8 eyes) and (b) oGVHD (n = 7, 14 eyes). This sample size will allow detection of a group mean difference of 1.26 standard deviation for a power of 0.8 and a significance level of 0.05.

Classification of patients was determined as follows: the control group included individuals with no ocular symptoms, a Schirmer I test without anesthesia result exceeding 10 mm at 5 minutes and an Ocular Surface Disease Index (OSDI) score below 13. Patients were considered to have ADDE if their Schirmer I at 5 minutes was less than 10 mm of wetting, vital dye staining of the cornea ≥1 on the NEI-CSS, and an OSDI > 13^45,46^. Schirmer I testing is a standard test used as an element in the diagnosis of ADDE^47,48^. SS diagnosis was based on a preexisting diagnosis from a board certified rheumatologist with either presence of specific serum auto-antibodies or the presence of a disease consistent biopsy and concomitant ADDE as noted above. oGVHD diagnosis was based on previously reported consensus guideline criteria^49^. Briefly, it includes 4 subjective and 4 objective criteria, corneal staining OSDI, Schirmer I,tear production measurements, and conjunctival injection. All patients in the oGVHD group that were included in the study had a diagnosis of “definite” oGVHD.

Examination and testing order during clinical visits was performed in the following order: OSDI scoring, Schirmer I, slit lamp biomicroscopic examination of the ocular surface (using a broad beam at medium intensity), ocular surface washings/tear collection, Vital dye staining and evaluation of the ocular surface with fluorescein.

### Schirmer I (without anesthesia) testing and Ocular Surface Disease Index (OSDI)

Schirmer I testing was undertaken to measure tear production using Whatman filter strips #41 (Haag-Streit, Essex, UK). After 5 minutes, the Schirmer strip was removed and the length of wetting (mm) was measured. OSDI scores range from 0–100 with higher scores associated with more severe DED or disability. OSDI score was calculated using the following formula, OSDI = [Σ(scores of all the questions) × 25]/(total number of questions)^20,21^.

### Corneal staining

Corneal staining score as measured by Lissamine Green dye staining (5 μL of 1% solution applied to each eye) using National Eye Institute-Corneal Staining score (NEI-CSS), grading scale^46,50^. The NEI scale relies on a chart that divides the cornea into five sections and assigns a value from 0 (absent) to 3 (severe) to each section, based on the density of punctate keratitis, for a maximum of 15 points.

### Non-invasive tear breakup time (NITBUT)

NITBUT is the time (in seconds) it takes for distortions to appear in the image of concentric Placido rings that are reflected on the patient’s cornea by a keratograph (Oculus, Inc., Arlington, WA). Two types of NITBUT are measured by the Keratograph 5 M: (i) NITBUT-first is the time at which the first distortion of Placido rings occurs; and (ii) average NITBUT-is the average time of first breakup incidents in different locations in a corneal diameter of 8 mm. We have provided information on the average NITBUT to compare the tear breakup time amongst ADDE subgroups (oGVHD and SS) (Supplementary Table 1).

### Meibomian gland analysis

Meibomian Gland imaging was performed using LipiView II Ocular Surface Interferometer (TearScience, Morrisville, NC). Meibomian gland dropout was graded using a 0 to 4 scale based on the area of Meibomian gland loss (0, 0%; 1, <25%; 2, 25%–50%; 3, 51%–75%; and 4, >75%). The score was recorded as “Meiboscale” for each eye^23,24^.

### Tear collection

Ocular surface washings (OSW) were performed for tear collection, following published protocols^20,43^. OSW were preferred, as opposed to direct capture of tears with microcapillary tube, as sufficient tear volume was not present to directly collect tears from patients with ADDE. In order to maintain comparability the same method of collection for tears was performed for normal patients. Briefly, ocular surface washings were obtained using a 50 µl drop of preservative-free artificial tears (Refresh Optive Sensitive; Allergan, Inc.) instilled in the eye by pipette, and OSW were performed then after a 2-minute period, tears from the lower lid margin and inferior fornix of all patients using a blunt glass microcapillary tube (5-μL Drummond Microcaps, Cat.#21-170E; Thermo Fisher Scientific, Waltham, MA) were collected. OSW tears were stored in a sterile Eppendorf tube [Thermo Fisher (Cat.#9501)] at −80 °C^20^.

### Saliva collection

Unstimulated whole saliva was collected from normal subjects without known oral diseases including severe periodontitis or dry mouth. Saliva was collected similarly to published protocols^51,52^. Briefly, without any stimulation, saliva is allowed to accumulate in the mouth and expelled into a sterile culture dish. Approximately, 2 ml of whole saliva was collected and centrifuged at 14000 RPM at 4 °C for 10 minutes; the supernatant was then used for experimentation. Saliva was collected only for use as a positive biological control to determine the presence or absence of H1 in tears (Figs 1A and 2).

### Histatin-1 synthesis

Histatin-1 peptide was synthesized according to previous published protocol as Trifluoroacetic acid (TFA) salts^30^. The sequence of the peptide was, DS(PO3)HEKRHHGYRRKFHEKHHSHREFPFYGDYGSNYLYDN with phosphorylation of ‘S (Serine)’ and without any other modification. The theoretical size of H1 is 4928.17. Briefly, standard solid phase using Fmoc chemistry was used for the H1 synthesis. The peptide was purified using HPLC and characterized by electrospray ionization mass spectrometry. Lyophilized peptide powder was dissolved in phosphate buffered saline (PBS) to use for all the experiments. H1peptide was synthesized as Trifluoroacetic acid (TFA) salts with >95% purity and without any modification.

### Western Blot (WB), immuno- dot blot (IDB) and coomassie staining

WB was performed following standard methods^53^. Briefly, tear samples (2.5 µl) were electrophoresed on 12% NUPage bis-Tris gels (Invitrogen, Carlsbad, CA) and transferred to nitrocellulose membranes. Membranes were blocked with Tris-buffered saline containing 3% nonfat dry milk and 0.02% Tween 20 and incubated with 1:1000 rabbit anti- human Histatin-1 (H1) antibody (MBS2002621, Mybiosource, San Diego, CA) overnight at 4**°**C and with 1:2000 goat anti-rabbit-HRP (BD Biosciences, San Jose, CA) as the secondary antibodies for 1 h. The membranes were developed using X-ray film and ECL Pro solution (PerkinElmer, Waltham, MA). For WB protein quantification was performed using BioDrop (Biochrom Ltd, Cambridge, UK). For IDB, 4 µl of serial dilutions of synthetic H1 (Syn.H1) were spotted in a nitrocellulose membrane and dried. Syn.H1 peptide and saliva were used as positive controls and a scrambled peptide and PBS were used as a negative control. Rabbit anti- human H1 antibody or Mouse anti- human H1 antibody (ab 70024, Cambridge MA, Anti-Histatin 1) were used for primary antibodies. For detection, Goat anti-Rabbit (IRDy@ 680 RD) or Goat anti-mouse (IRDy @ 800 Cw) were used as secondary antibodies. The relative intensity of each band was determined with the Li-Cor Odyssey application software (LiCor Biosciences). For qualitative comparison of the level of total protein between tear samples, we stained gels with Coomassie Brilliant Blue R-250 staining solution (Bio-rad Hercules, California, USA) as has been performed for qualitative comparison of protein levels in tears^44^.

### Multiple reaction monitoring (MRM)

MRM was performed for identification of presence of H1 in tears on the LC–MS/MS system (Agilent 1200 HPLC (Agilent Technologies, Santa Clara, CA) coupled with mass spectrometry (QTRAP 6500 Sciex, Framingham, MA)^54^. Chromatography was performed using a binary solvent system consisting of A: 0.1% formic acid and 5% ACN and B: 0.1% formic acid and 95% ACN at a flow rate of 200 μL/min and mobile phase and reversed-phase columns SB-C18 (2.1 mm × 50 mm, particle size 1.8 µm, Agilent Technologies). A gradient was run from 5% B to 60% B over 10 min. A standard curve for H1 was constructed using a range of concentrations with (Syn.H1. Samples were centrifuged at 14,000 RPM for 9 minutes followed by injection of 10 µL of the supernatant into the LC-MS/MS system. The MRM was operated in the positive ion mode monitoring the 704.9–931.0 m/z, transitions for H1.

### Enzyme-linked immunosorbent assay (ELISA)

H1 concentration was determined by ELISA detection using a commercially available H1 ELISA kit (MBS2022124 H1, MyBioSource, Inc.,San Diego,CA, USA) following the manufacturer’s instructions and read using a microplate reader (Biotek Synergy H1,Winooski WI), and published protocols^55^. Recent studies on salivary histatin and other proteins levels have been identify/quantify using ELISA kit (MyBioSource,Inc.)^56^.

### Statistical analysis

The data were summarized by means and standard errors for continuous variables or by proportions for categorical variables. Demographic characteristics (age, sex) were compared between normal and ADDE groups using t-tests or Chi-square tests. The values of Schirmer I, OSDI and H1 concentration from two eyes were averaged first for each participant then were compared between groups using t-tests. The association between H1 concentration and other measures (age, sex, Schirmer I, OSDI and disease status) were assessed using mixed linear models, which could account for between eye correlation. Finally, logistic regression was used to estimate OR of disease from the averaged H1 levels from two eyes for each individual, followed by ROC analysis.

## Supplementary information

Supplementary data
