# Supplementary material for: Presence of Histatin-1 in Human Tears and Association with Aqueous Deficient Dry Eye Diagnosis: A Preliminary Study
Source: Sci Rep. 2019 Jul 16;9:10304. doi: 10.1038/s41598-019-46623-9 (PMC6635511; doi:10.1038/s41598-019-46623-9)
Supplement: Supplementary file 1 — Supplementary data [file 41598_2019_46623_MOESM1_ESM.docx]

**Title**

**Presence of Histatin-1 in Human Tears and Association with Aqueous Deficient Dry Eye Diagnosis: A Preliminary Study**

Sushma Kalmodia, PhD*^1^, Kyung-No Son, PhD*^1^, Dingcai Cao, PhD^1^, Bao-Shiang Lee, PhD^2^, Dhara Shah, MS^1^,Marwan Ali, MRes^1^, Arun Balasubramaniam, PhD^1^, Sandeep Jain, MD^1^, Vinay Kumar Aakalu, MD MPH^1^

*These authors contributed equally to this study and manuscript preparation

Corresponding Author:

Vinay Kumar Aakalu, MD MPH1

1855 W. Taylor St.

MC 648, Suite 3.158

Chicago, IL 60612

vaakalu@uic.edu

Institutions

1 Department of Ophthalmology and Visual Sciences, University of Illinois at Chicago

2 Research Resources Center, University of Illinois at Chicago

**Supplementary Figure**


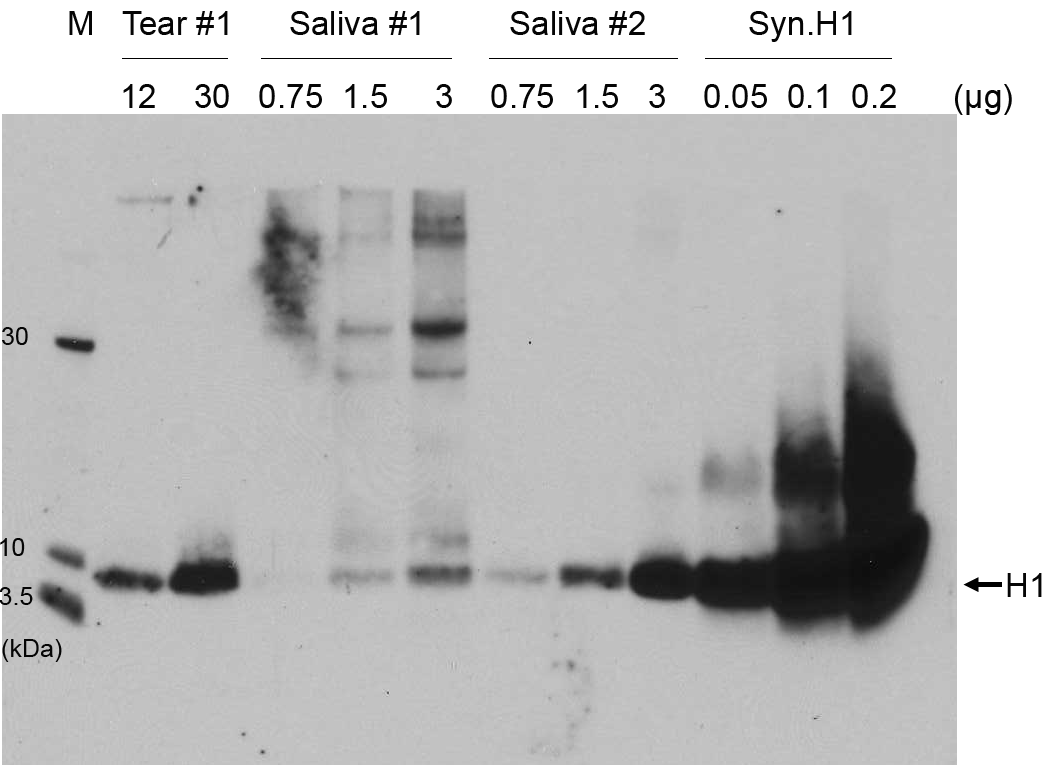


**Fig S1.**  Full length Western Immunoblotting (WB) image developed on X-ray film.

**M** (Molecular size marker)

**H1** (Histatin-1)

**Supplementary Table-1:** ADDE subgroup (oGVHD and SS) Patient Phenotypic Characteristics

| **Risk Factors** | **SS**  (mean±SEM) | **oGVHD**  (mean±SEM) | **p-value** |
| --- | --- | --- | --- |
| **Schirmer I (mm))** | 3.2±2.1 | 1.1±0.0 | 0.231 |
| **OSDI (0-100)** | 48.1±12.3 | 25.6 ±6.2 | 0.099 |
| **Corneal Staining Score (CSS) (0-15)** | 2.4±1.0 | 4.8±1.6 | 0.3045 |
| **Average NITBUT (secs)** | 4.1±1.3 | 5.5±0.8 | 0.392 |
| **Meiboscale score (0-4)** | 0.4±0.2 | 1.1±0.1 | 0.0202 |
| **H1 (ng/ml)** | 197.6±170.2 | 22.0±22.0 | 0.199 |

**ADDE-** aqueous deficient dry eye disease

**SS-** Sjögren’s syndrome

**oGVHD-** ocular Graft-versus-Host Disease

**OSDI-** Ocular Surface Disease Index

**CSS-** Corneal Staining Score

**Schirmer I (mm)-** Schirmer I ( without anesthesia)

**NIBUT-** Non-invasive tear break-up time

**H1**- Histatin-1
